# Anthropometric parameters as a tool for the prediction of metabolic and cardiovascular risk in childhood brain tumor survivors

**DOI:** 10.1186/s13098-024-01262-7

**Published:** 2024-01-19

**Authors:** Alberto Romano, Mariapia Masino, Serena Rivetti, Stefano Mastrangelo, Giorgio Attinà, Palma Maurizi, Antonio Ruggiero

**Affiliations:** 1 Pediatric Oncology Unit, Fondazione Policlinico Universitario Agostino Gemelli IRCCS, 00168 Rome, Italy; 2 Università Cattolica del Sacro Cuore, 00168 Rome, Italy

**Keywords:** Brain tumor, Metabolic syndrome, Cardiovascular risk, Childhood cancer survivor

## Abstract

**Purpose:**

To assess the prevalence of alterations in anthropometric parameters predictive of metabolic syndrome and cardiovascular risk among childhood brain tumor survivors.

**Methods:**

Anthropometric parameters predictive of metabolic syndrome and cardiovascular risk were analyzed [height, weight, BMI, waist circumference, hip circumference, waist-height ratio (WHtR), waist-hip ratio (WHR, blood pressure] of 25 patients who survived childhood brain tumors.

**Results:**

21 patients (84%) showed alteration of at least one predictive anthropometric parameter. 11 patients (44%) showed a BMI > 75th percentile and 19 patients (76%) showed a pathological WHR value. A pathological WHtR (> 0.5), was identified in 17 patients (68%); the average WHtR observed was 0.53. 9 patients (36%) showed an alteration of all three anthropometric parameters considered. Comparing this subpopulation with the subpopulation with less than three altered parameters, a greater prevalence of the combined alteration was observed in the female sex compared to the male sex (67% vs. 26%). No significant differences were observed regarding the age of diagnosis and end of treatment nor the treatments carried out (chemotherapy, radiotherapy, steroid therapy) between the two groups.

**Conclusion:**

These results suggest that this population is at high risk of presenting pathological values of BMI, WHR and WHtR with consequent high risk of developing metabolic syndrome and cardiovascular diseases.

## Introduction

Central nervous system (CNS) tumors are the second most frequent neoplasm in pediatric age, after leukemia, accounting for approximately 25% of all tumors [1]. Treatment of brain tumors is individualized and depends on tumor type, extent, location, molecular features, age, and associated symptomatology and involves the integration of surgery, chemotherapy, and radiation therapy [2].

Over the years, brain tumor survival has increased; to date, about half of children with brain tumors recover and become adults. This has allowed attention to be focused on the long-term effects of cancer treatments including neurocognitive deficits, endocrinological sequelae, reduced statural growth, ototoxicity, renal failure, cataracts, infertility, sarcopenia, and secondary malignancies [3–8].

Treatments used for the management of brain tumors, in addition to having major sequelae in several organs (including kidney, heart, and endocrine system), also cause activation of chronic inflammatory processes and metabolic alterations that can promote the onset of metabolic syndrome and increased cardiovascular risk [9–11].

Radiation therapy, in addition to acting locally by promoting tumor destruction, can cause dysfunction through the involvement of surrounding healthy structures. In particular, when carried out in the brain, it can cause alteration of the hypothalamic-pituitary axis resulting in a change in body fat distribution, with an increase in the ratio of android to gynoid fat [10]; in addition, high-dose radiation (> 30 Gy) results in a possible development of leptin resistance at hypothalamic receptors [12] and overproduction of it by adipose tissue [13]. The failure of leptin action on the hypothalamus causes an increase in hunger and food intake, which in turn results in increased adipose tissue and the appearance of insulin resistance [14].

Also, radiation therapy at the hypothalamic level may result in GH deficiency, with increased lipid and free fatty acid oxidation that is associated with insulin resistance, central obesity, and dyslipidemia [15]. All this contributes to the development of metabolic syndrome and the onset of cardiovascular disease in patients survived to childhood brain tumors.

In addition, patients with brain tumors often require prolonged steroid therapies leading to an increased risk of the occurrence of diabetes mellitus, cytokine release, and adipose tissue accumulation that can lead to hepatic steatosis, atherosclerosis, and thrombosis [16, 17].

The mechanisms described cooperate generating a chronic inflammatory state, metabolic syndrome and increased cardiovascular risk with significant effect on the health of childhood cancer survivors. Indeed, it has been shown that deaths from cardiovascular causes are 7 times more frequent in childhood cancer survivors than in the general population, accounting for a quarter of all deaths within 45 years of cancer diagnosis [18]. Such high incidence explains the need for close monitoring of childhood cancer survivors to diagnose metabolic syndrome early and reduce cardiovascular risk.

The metabolic syndrome is not a single disease but a constellation of risk factors for cardiovascular disease such as the presence of insulin resistance (impaired fasting blood glucose, impaired glucose tolerance, or type 2 diabetes mellitus), in addition to two of the following risk factors: obesity, hyperlipidemia (hypertriglyceridemia, low HDL), hypertension, or microalbuminuria [19].

Despite the increasing prevalence of obesity and its metabolic complications in the pediatric population [20], to date there is still no general consensus on how to define metabolic syndrome in children and adolescents, although there have been several attempts to modify the adult diagnostic criteria and use them for the pediatric population [21].

Over the years, it has been observed that certain anthropometric parameters, alone or in combination, can identify groups of patients at risk for developing metabolic syndrome and increased cardiovascular risk. Specifically, obesity is characterized by excessive accumulation of fat mass in adipose tissue, subcutaneous tissue, and within organs. The definition of overweight and obesity is based on body mass index (BMI), defined as the ratio of weight (in kg) to the square of height (in m). Although BMI is easy to apply, it has a number of critical issues, including estimating excess weight rather than excess fat [22].

The risk, however, lies less in the amount of fat in the body and more in its distribution.

Body fat distribution can be estimated through parameters such as waist circumference and waist-to-height ratio [23].

The use of these anthropometric parameters, or a combination of them, although not allowing a diagnosis of metabolic syndrome, can help in metabolic and cardiovascular risk stratification [22].

## Objectives of the study

Primary objective:

- To assess the prevalence of alterations in anthropometric parameters predictive of metabolic syndrome and cardiovascular risk among childhood brain tumor survivors.

Secondary objective:

- To compare the characteristics of the population considered at risk for metabolic syndrome and cardiovascular events (for alteration of 3 anthropometric parameters) and the population with less than 3 factors.

## Materials and methods

The following is a prospective observational study, inherent to the analysis of predictive factors of occurrence of metabolic syndrome and cardiovascular risk in pediatric brain tumor survivors.

The study was conducted on a group of 25 patients who had survived childhood brain tumors and were in follow-up at the Pediatric Oncology Unit of the Foundation Polyclinic Agostino Gemelli IRCCS. Patients were selected respecting the following inclusion criteria:

- Brain tumor diagnosis made between the ages of 0 and 21 years;
- Age at the time of enrollment greater than or equal to 10 years;
- Having received radiotherapy associated with chemotherapy or not;
- Remission of disease for at least 5 years evidenced by at least one negative MRI examination for the presence of disease in the past 12 months or remission of disease for at least 10 years even in the absence of an MRI examination in the last 12 months;
- Absence of known and ongoing cardiac disease at the time of enrollment;
- Absence of congenital heart disease.
- Karnofsky Performance Status Index > 90.

Exclusion criteria were:

- Remission of disease for less than 5 years;
- Age less than 10 years at the time of enrollment.

All subjects enrolled at the time of evaluation underwent:

- General medical history (age, sex, origin, current relevant diseases and treatments, hormone replacement therapy, physical activity performed);
- Remote pathological history, with data related to neoplastic pathology (tumor location, histology, date of diagnosis, disease staging, anthropometric data at diagnosis, type and duration of therapy performed, date of end of treatment, any steroid therapy during treatment).

Measurement of anthropometric data was also performed for each patient:

- Height, height-for-age centile, Z-score. Centiles were calculated with a percentile calculator available on the Internet, based on CDC growth charts [24].
- Weight, centile weight-for-age, Z-score. Weight was measured in the absence of clothing (except undergarments) with electronic scales. Centiles were calculated with a percentile calculator available on the Internet, based on CDC growth Table [24].
- BMI, centile BMI-for-age, Z-score. BMI was calculated using the formula (weight in kg)/(height in m)2. The percentile and Z-score BMI were calculated by percentile calculator available on the Internet, based on CDC growth Table [24]. In accordance with SIEDP (Italian Society of Diabetology and Child Endocrinology) guidelines, patients with BMI > 75th percentile and obese patients with BMI > 95th percentile were considered overweight [25, 26].
- Waist circumference, measured at the point of smallest circumference between the last rib and the top of the iliac crest and reported in cm.
- Hip circumference, measured at the major circumference point at the posterior extension of the buttocks and reported in cm.
- WHR, measured using the formula (waist circumference in cm)/(hip circumference in cm); based on the value obtained, tables were compared to establish cardiometabolic risk, as reported in “Obesity. Part I-Pathogenesis” (Table 1) [27].
- WHtR, measured by the formula (waist circumference in cm)/(height in cm); a value of WHtR > 0.5 was considered indicative of central obesity [28].
- Systolic and diastolic blood pressures, reported in mmHg and pressure-per-age percentiles, calculated for patients > 18 years by comparison of CDC Table [29], for patients < 18 years by calculator available on the internet [30].

**Table 1:** Cardiometabolic risk based on WHR

	Age	Low	Moderate	High	Very high
**Women**	20–29	< 0,71	0,71 − 0,77	0,78 − 0,82	> 0,82
	30–39	< 0,72	0,72 − 0,78	0,79 − 0,84	> 0,84
	40–49	< 0,73	0,73 − 0,79	0,80 − 0,87	> 0,87
	50–59	< 0,74	0,74 − 0,81	0,82 − 0,88	> 0,88
	60–69	< 0,76	0,76 − 0,83	0,84 − 0,90	> 0,90
**Male**	20–29	< 0,83	0,83 − 0,88	0,89 − 0,94	> 0,94
	30–39	< 0,84	0,84 − 0,91	0,92 − 0,96	> 0.96
	40–49	< 0,88	0,88 − 0,95	0,96 − 1,00	> 1.00
	50–59	< 0,90	0,90 − 0,96	0,97 − 1,02	> 1.02
	60–69	< 0,91	0,91 − 0,98	0,99 − 1,03	> 1.03

Based on BMI, WHR and WHtR, we divided the patients studied into two groups: at-risk population (with pathological values of all three indicators) and non-at-risk population and compared their characteristics.

Given the absence of data on early indicators of metabolic syndrome in the population of interest, we set the sample size for this study at 25 patients.

This numerosity, however, manages to intercept various expected proportions, with a 95% confidence level and a margin of error ranging from a minimum of 3.9% (for an expected proportion of 1%) to a maximum of 19.6 (for an expected proportion of 50%).

Quantitative variables were presented through mean and standard deviation (SD), qualitative variables through absolute and percentage frequency tables.

Comparison of means was performed by Mann-Whitney test, comparison of proportions by chi-square test, which was considered statistically significant for p-values < 0.05. Statistical evaluation was performed by XLSTAT 2023.1.4.1408.

## Results

The population sample taken into analysis was 25 patients, including 19 males (76%) and 6 females (24%), whose brain tumor diagnosis was made between August 2003 and October 2015. Table 2 shows the main demographic and anthropometric characteristics of the patients enrolled at the time of diagnosis.

**Table 2:** Demographic and anthropometric characteristics of patients at diagnosis

	Mean	SD
Age (years)	8,84	6,23
Weight (kg)	40,648	25,401
Centile weight for age	66,217	34,076
High (m)	1,320	0,315
Centile high for age	50,000	33,694
BMI (Kg/m2)	21,245	5,878
Centile BMI for age	73,524	35,688

SD: standard deviation; BMI: body mass index

44% of the population examined had a diagnosis of germinoma, 32% medulloblastoma, 16% ependymoma, and 8% glial neoplasm.

The primary tumor location was divided according to the involved cranial fossa. Table 3 shows the main clinical features of the tumor at diagnosis.

**Table 3:** Characteristics of the tumor at diagnosis

	Number of patients (%)
Localization of the tumor	
Anterior cranil fossa	1 (4)
Middle cranial fossa	12 (48)
Posterior cranial fossa	12 (48)
Histology	
Germinoma	11 (44)
Medulloblastoma	8 (32)
Ependimoma	4 (16)
Glial tumor	2 (8)

Patients, based on tumor diagnosis, histological features and staging, were treated according to various protocols, most of which consider the combination of chemotherapy and radiotherapy. Table 4 shows the main features of chemotherapy treatment.

**Table 4:** Characteristics of chemotherapy treatment

Chemotherapy	Number of patients	%	Mean (mg/m2)	SD
Carboplatin	11	44	1350,5	704,6
Cisplatin	9	36	362,4	256,7
Ifosfamide	9	36	38458,3	11834,5
Cyclophophosphamide	6	24	3375,8	4278,6

SD: standard deviation

12% (2 patients) received high-dose chemotherapy and autologous hematopoietic stem cell transplantation.

56% (14 patients) received cortisone for more than 14 days.

All 25 were treated with radiotherapy and irradiated at encephalic level: of them, 6 (24%) received RT on marrow, with dose of 30 Gy.

Of 25 total patients, 20% (5) had disease recurrence.

The evaluated population finished treatment between May 2004 and January 2017 (including treatment of relapses). At the time of evaluation, the mean age of the population taken into analysis was 22.4 years, with SD of 7.49.

The anthropometric parameters analyzed, were collected at a minimum distance of 5 years after the end of treatment, shown in the Table 5.

**Table 5:** Anthropometric parameters at the time of enrollment

	Mean	SD
Weight (kg)	68,64	23,79
Weight centile for age	55,83	30,75
High (m)	1,63	0,16
High centile for age	32,52	31,52
BMI (Kg/m2)	25,3	6,8
BMI centile for age	65,75	27,02
Waist circumference	86,7	18,01
Hip circumference	98,09	16,12
WHtR	0,53	0,106
WHR	0,88	0,096
Sistolic blood pressure (mmHg)	111,86	6,94
Diastolic blood pressure (mmHg)	88,21	9,39

SD: standard deviation; WHtR: waist to high ratio; WHR: waist to hip ratio

In addition, the presence of endocrine deficit and the level of physical activity performed regularly by the patients were also researched. Following treatment, 11 patients (44%) had no endocrine deficit, 5 patients (20%) developed hypothyroidism, 4 patients (16%) developed GH deficiency, and 5 patients (20%) developed panhypopituitarism. Of the 25 patients, 15 (60%) performed physical activity regularly, while 10 (40%) were inactive.

Anthropometric parameters related to body fat distribution and obesity were also considered. The main parameters examined were BMI, the ratio of abdominal circumference to hip circumference (WHR), and the ratio of abdominal circumference to height (WHtR).

Regarding BMI, in accordance with the SIEDP definition, there were 11 patients who showed a BMI > 75th percentile, thus pathological, (44% of the total).

The WHR value was pathological by sex and age in 76% of patients (19 patients). The remaining 24% were found to be nonpathological. According to Table 6, based on the WHR value, the relative risk of incidence of cardiovascular and metabolic diseases was divided [8]: of 25 patients, 5 (20%) were found to be at low risk, 5 (20%) at moderate risk, 5 (20%) at high risk, and 10 (40%) at very high risk.

In relation to WHtR, the value considered pathological is > 0.5. Of the total 25 patients, 8 (32%) presented a WHtR within normal limits and 17 (68%) showed a pathological value.

Because these 3 parameters are considered to be excellent predictors of cardiovascular and metabolic risk, a sample of the patients with BMI > 75th percentile, pathological WHR and WHtR > 0.5 was selected and compared with the rest of the population.

The sample under analysis consisted of 9 patients (36% of the total), of whom 4 were female (44.4%) and 5 were male (55.5%).

**Table 6:** Comparison between the at-risk population and the not-at-risk population

	At-risk population		Not-at-risk population		
	Mean/Number of patients	SD	Mean/Number of patients	SD	p-value
Age at diagnosis	10,22	7,293	8,06	5,651	0,619
Age at end of treatment	12,23	7,543	9,96	5,060	0,568
Female sex	4	44,44	2	12,50	**0,037**
Male sex	5	55,56	14	87,50	
Regular physical activity	5	55,56	10	62,5	0,932
Histology					
Germinoma	3	33,33	8	50,00	**0,011**
Medulloblastoma	4	44,44	4	25,00	0,420
Ependimoma	1	11,11	3	18,75	0,910
Glial tumor	1	11,11	1	6,25	0,667
Localization of the tumor					
Anterior cranial fossa	1	11,11	0	0	0,173
Middle cranial fossa	3	33,33	8	50	0,420
Posterior cranial fossa	5	55,56	8	50	0,789

Regarding demographic characteristics, according to age of diagnosis and end of treatment, no statistically significant differences were found. No statistically significant differences were found according to physical activity.

In contrast, regarding sex, a statistically significant difference was identified for the female sex; in fact, about 67% of the total female population presented with 3 risk factors, compared with 26% of the total male population.

Relative to tumor characteristics, 33.3% of the sample taken into analysis have a diagnosis of germinoma, 44.4% medulloblastoma, 11.1% ependymoma, and 11.1% glial neoplasm.

From a localization point of view, 5 are at the level of the posterior cranial fossa, 1 at the level of the anterior cranial fossa and 3 at the level of the middle cranial fossa, presenting no significance.

Considering the chemotherapy performed, no significant differences were demonstrated between patients with all 3 risk factors and patients with less than 3 risk factors.

The cranial RT dose in patients with 3 risk factors was 49.1 Gy, with a SD of 7.756; in non-risk patients, the average dose administered was 46.6 Gy with a SD of 13.728. The p-value of 0.936 was non significant. Spinal RT was performed in 3 patients at risk (33.3%), while it was not performed in 6 (66.7%), with a p-value of 0.412.

## Discussion

In recent years, improved treatments for childhood cancers and the adoption of international protocols-with the combination of surgery, radiotherapy and chemotherapy-have led to increased survival; with it, long-term treatment-related side effects have been observed [18]. In particular, cranial radiation affects the hypothalamic-pituitary axis, which may lead to growth hormone deficiency and leptin insensitivity, which could, in turn, put brain tumor survivors at risk for neuroendocrine diseases such as obesity [31].

Obesity is a key component of the metabolic syndrome; specifically, visceral obesity, regardless of the method of measurement, is associated with an increased incidence of metabolic diseases (diabetes and/or glucose intolerance, dyslipidemia, hyperuricemia), cardiovascular diseases (hypertension, ischemic heart disease and heart failure) or systemic diseases (osteoarthritis, colon cancer, respiratory failure, cholelithiasis, etc. ), while in the subcutaneous or peripheral one, the incidence of such pathologies is less evident [32]. In addition, it is important to consider that obesity is a reversible disease condition, which can be acted upon through weight and BMI reduction.

The incidence of Metabolic Syndrome increases with increasing age, however, it has been shown by several studies that the prevalence in pediatric cancer survivors is higher than that estimated in the healthy control population [33].

Previous studies have shown the early onset of serious cardiac events (such as heart failure, coronary artery disease, and cerebrovascular events) in pediatric cancer survivors with metabolic syndrome, often leading to early mortality [31]. In fact, death from cardiovascular causes is seven times more frequent in this group of patients than in the general population and accounts for a quarter of all deaths within 45 years of cancer diagnosis. Such a high incidence explains the need for close monitoring childhood cancer survivors to detect early risk factors for metabolic syndrome and reduce cardiovascular risk.

Our study analyzed 25 pediatric brain tumor survivors, assessing the prevalence of anthropometric changes predictive of cardiometabolic pathology following radiotherapy associated or not with chemotherapy; it involved a population of 19 males and 6 females, with an age at assessment ranging from 10 years to 41 years. The evaluation was performed a minimum of 5 years after the end of treatment.

The present study showed a high prevalence of anthropometric alterations in the population taken into analysis: out of 25 patients analyzed, 21 (84%) showed alteration of at least one predictive anthropometric parameter.

46% of patients showed alteration regarding BMI, compared with 42.9% observed in the Italian populatio. In line with the data we analyzed, in the study by Wang et all, the rate of overweight and obesity in patients treated for brain tumor is 42.6%, 31.70% if we exclude patients with craniopharyngioma and 40.4% of noncancerous controls [34, 35]. In contrast, in the study of Belle et al. [36], considering childhood cancer survivors, the prevalence of overweight and obesity is comparable to that of the general population (about 22% compared with 25% in the general population). This is true for most childhood cancers such as leukemia (26%), neuroblastoma (13%) or soft tissue sarcoma (18%), in contrast to brain tumor patients (31%), who, also in the study by Belle et al. have a significantly higher prevalence of overweight and obesity [36].

Relative to WHR, it was shown to be pathological in 76% of the population examined, compared with a prevalence of 59.5% of the Italian population analyzed in The CUORE project study [37]. The overall mean WHR of our population is 0.88, in line with results reported by Wang et al., who demonstrated a mean WHR in brain tumor survivors is 0.87 [35].

Similarly, WHtR in our population was found to be increased in 68%, concordant with the data reported in the study by Karlage et al. in which the prevalence in childhood cancer survivors is about 62.5% [38]. In the study by Steinberger et al., a prevalence of 24% was reported in the population of childhood cancer survivors compared with 11.2% in healthy controls [39]. The mean WHtR in our population is 0.53; in other studies a mean value of 0.48 has been reported, while in the general population the mean is about 0.43.

These results indicate that the brain tumor survivors included in our study have significantly higher total and central adiposity than the non-cancer population and also than survivors of tumors other than brain tumors [34].

9 patients (36% of the total) showed concomitant alteration of the 3 risk factors considered; a comparison of characteristics was made between the subpopulation with 3 risk factors and the population with less than 3 risk factors. No statistically significant differences were observed for age at diagnosis (p-value: 0.619) and age at end of treatment (p-value 0.568), unlike the study by A. Agarwal et al. [31], which showed that older age at diagnosis was an independent predictor of metabolic syndrome.

Regarding sex, the prevalence of female patients with the alteration of 3 risk factors was 67%, compared with 26% of male patients. Female sex was shown to be a statistically significant risk factor for obesity, consistent with a report conducted on pediatric cancer survivors, which showed that female patients undergoing > 20 Gy cranial RT had an increased risk of obesity during adult life [34]. This, however, is in contrast to Wang’s systematic review and meta-analysis, which defined the greater likelihood of men to be overweight than women (OR 1.8 95%) [35]. In the reviewed literature, therefore, the role of gender as a risk factor is still unclear. In our series, female sex appears to be a risk factor and we can hypothesize that this is linked to more complex hormonal disorders involving the hypothalamic pituitary axis and the production of gonadotropins and their peripheral effectors (estrogen, progesterone and testosterone). However, further studies including laboratory investigations would be necessary to better investigate this aspect.

When analyzing tumor characteristics, the site of primary location did not significantly influence the development of risk factors.

Regarding the treatments carried out, no statistically significant differences were identified for chemotherapy, radiotherapy or steroid therapy in the two groups examined, probably due to the homogeneity in the treatments carried out.

However, since the prevalence of obesity and visceral adiposity appears to be very high in our population compared to the general population and/or the prevalence reported in the studies cited above (BMI 46% vs. 42.9%, WHR 76% vs. 59.5%, WHtR 68% vs. 11.2%), we can suppose that RT may be a risk factor in the development of obesity, as demonstrated by Belle et al. [36].

The primary mediator of metabolic syndrome associated with cranial radiotherapy is growth hormone deficiency leading to obesity and dyslipidemia. In fact, hypothalamic lesions can compromise satiety signals and alter insulin, leptin and ghrelin signaling resulting in hyperphagia. It is also hypothesized that decreased sympathetic tone reduces adipose tissue lipolysis and basal metabolic rate, contributing to weight gain [35].

The main limitation of our study is the limited number of patients and the lack of a control group matched for age and sex. However, a strength can be found in long-term follow-up. Furthermore, the study provides a valuable insight into possible risk factors in the development of metabolic syndrome in pediatric brain tumor survivors and considers a possible predictive role of anthropometric parameters, such as BMI, WHR and WHtR.

## Conclusion

In our study we observed that the alteration of anthropometric indices linked to overweight/obesity and the distribution of body fat appears to have a high prevalence in patients survived to pediatric brain tumors.

Furthermore, we have observed that female sex is a risk factor for the development of obesity, even if the mechanisms are not yet well known.

Monitoring BMI, WHR and WHtR during follow-up can be a useful, simple, and fast tool for the early identification of subjects at risk of developing metabolic syndrome and for implementing prevention strategies like nutritional and an adequate nutritional and physical activity plan.

## Data Availability

No datasets were generated or analysed during the current study.
